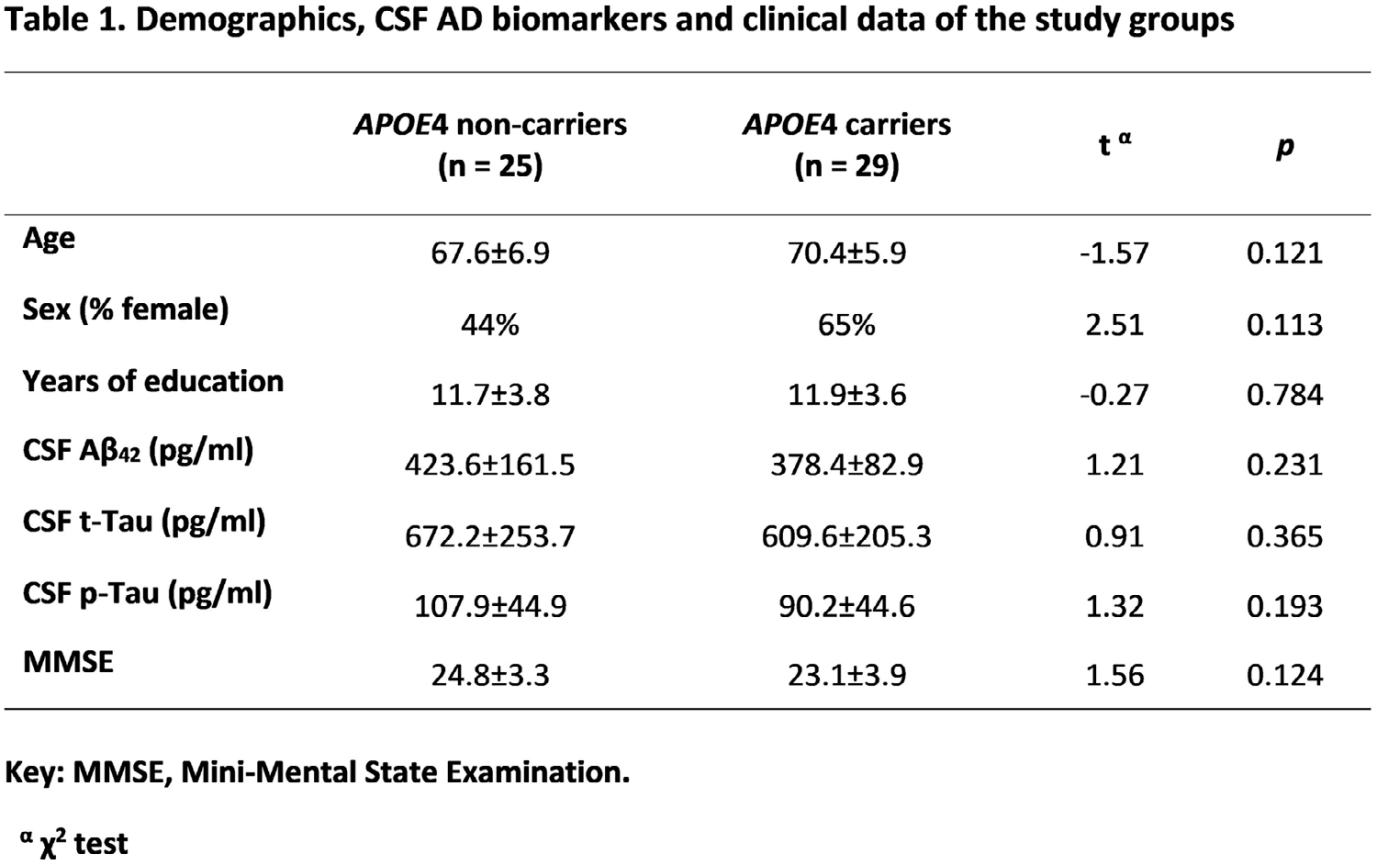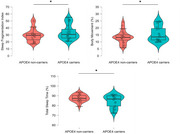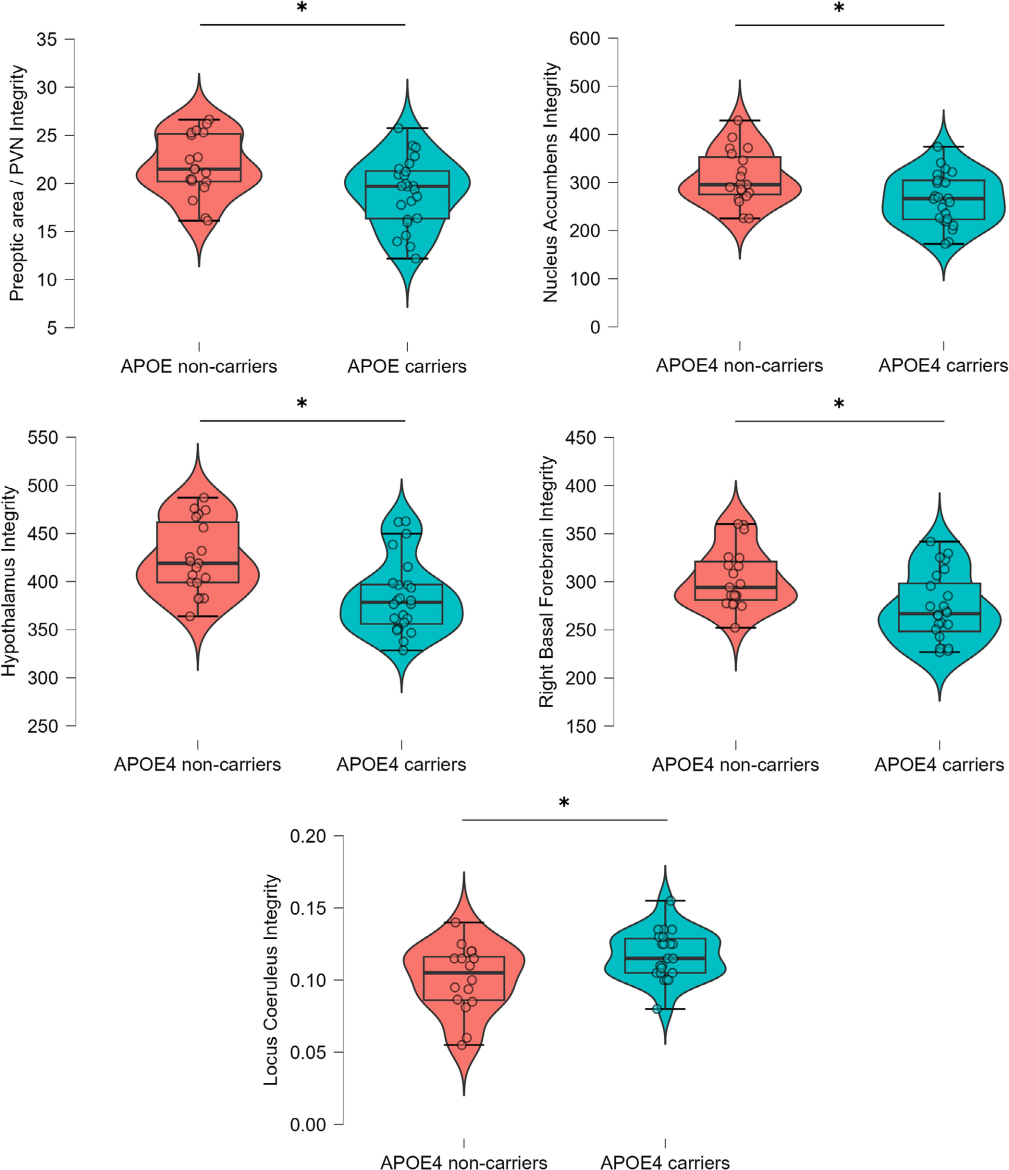# *APOE*4 status, sleep disturbances and neuromodulatory subcortical systems in Alzheimer's disease

**DOI:** 10.1002/alz70856_104909

**Published:** 2026-01-07

**Authors:** Adrià Tort‐Merino, Agnès Pérez‐Millan, Diana Esteller, Miquel Massons, Guadalupe Fernandez‐Villullas, Bea Bosch, Magdalena Castellví, Axel Rigol, Anna Antonell, Mircea Balasa, Albert Lladó, Raquel Sánchez‐Valle, Gerard Piñol‐Ripoll, Neus Falgàs Martínez

**Affiliations:** ^1^ Alzheimer's disease and other cognitive disorders Unit. Hospital Clínic de Barcelona. Fundació de Recerca Clínic Barcelona – IDIBAPS. University of Barcelona, Barcelona, Spain; ^2^ Centro de Investigación Biomédica en Red en Enfermedades Neurodegenerativas (CIBERNED), Madrid, Spain; ^3^ eHealth Center, Faculty of Computer Science, Multimedia and Telecommunications, Universitat Oberta de Catalunya, Barcelona, Spain; ^4^ Centro de Investigación Biomédica en Red en Enfermedades Neurodegenerativas (CIBERNED), Madrid, ‐, Spain; ^5^ Unitat Trastorns Cognitius, Clinical Neuroscience Research, Santa Maria University Hospital, IRBLleida, Lleida, Spain

## Abstract

**Background:**

Sleep alterations are common in Alzheimer's disease (AD) and may be related to the early degeneration of the neuromodulatory subcortical systems (NNS). The Apolipoprotein E‐ϵ4 (*APOE*4) allele is the major genetic risk factor of sporadic AD and has been associated with a faster rate of cognitive decline. Our aim was to study objective measures of sleep fragmentation and the NNS nuclei integrity in a sample of AD patients according to their *APOE*4 status.

**Method:**

We included 54 patients with a biomarker‐based diagnosis of AD, classified as *APOE4* non‐carriers (*n* = 25) and carriers (*n* = 29). Participants underwent clinical and neuropsychological evaluation, CSF extraction, blood sampling, 2‐week actigraphy (Motion Wath 8 device; CamNTech), and magnetic resonance imaging to measure NNS integrity. Analysis of variance (ANOVA) adjusted by age were used to compare the actigraphy measures and the NNS nuclei integrity between *APOE4* carriers and non‐carriers. In a subsample (*n* = 35), we run Repeated Measures ANOVA adjusted by age to explore longitudinal (1‐year follow‐up) neuropsychological performance among groups.

**Results:**

There were no significant differences between *APOE4* carriers and non‐carriers in terms of age, years of education, CSF AD biomarker levels or Mini‐Mental State Examination (MMSE) score (all *p*>0.05; Table 1). Compared to non‐carriers, *APOE4* carriers showed a higher sleep fragmentation index (F[1,41]=5.12; *p* <0.05), increased body movement (F[1,41]=4.61; *p* <0.05) and decreased total sleep time (F[1,36]=4.79; *p* <0.05) as measured by actigraphy (Figure 1)*. APOE4* carriers presented lower integrity (Figure 2) of the preoptic area and paraventricular nucleus (PVN) (F[1,40]=4.65; *p* <0.05), nucleus accumbens (F[1,40]=5.17; *p* <0.05), hypothalamus (F[1,40]=9.12; *p* <0.01), and the right basal forebrain (F[1,40]=6.10; *p* <0.05). On the contrary, *APOE*4 carriers showed higher locus coeruleus integrity (F[1,40]=5.12; *p* <0.05). While there were no between‐group differences in the neuropsychological scores at baseline, *APOE*4 carriers displayed a higher decline in verbal learning measures at the 1‐year follow‐up (F[1,29]=4.50; *p* <0.05).

**Conclusion:**

*APOE*4 carriers present higher sleep fragmentation and a higher vulnerability of several NSS nuclei when compared with non‐carriers. Further longitudinal research is called for to assess the relevance of the current findings as prognostic markers of the disease.